# Reducing the low-prevalence effect with probe trials

**DOI:** 10.1186/s41235-025-00702-w

**Published:** 2026-01-08

**Authors:** Mark W. Becker, Andrew Rodriguez, Derrek T. Montalvo, Chad Peltier

**Affiliations:** Department of Psychology, Michigan State University, Psychology Building, 31 Physics Dr., East Lansing, MI 48824 USA

**Keywords:** Low-prevalence effect, Visual search, Misses in visual search, Eye tracking

## Abstract

As targets become rare in visual search tasks, the likelihood of missing them increases—a phenomenon known as the low-prevalence effect (LPE). This has important implications for real-world searches, but reducing the LPE has proven challenging. In Experiment 1, we used a low-prevalence T-among-Ls task and found that distributing “probe” trials—trials with known targets and post-response feedback—reduced the LPE. In Experiment 2, participants searched for two low-prevalence targets (T and O among Ls and Qs), and we varied how often each appeared in probe trials. The probe benefit scaled with the frequency of the matching target, suggesting limited generalizability to non-probed targets. Experiment 3 used eye tracking to examine whether probes affected quitting thresholds, decision criteria, or guidance. Results showed that probes biased top-down guidance toward features of frequently probed targets, without affecting the number of items inspected or the decision criterion. In Experiment 4, we tested whether feedback was necessary for the probe benefit. Findings suggest that probes improve rare-target search by altering perceived prevalence, not through feedback alone. Overall, probes may reduce the LPE by increasing perceived prevalence and thereby increasing search guidance, but only when probe targets closely match actual search targets.

## Significance statement

When targets in a visual search task become rare, the likelihood of missing them increases greatly—the low-prevalence effect (LPE). Given that many important real-world search tasks (e.g., radiology, baggage screening) involve low-prevalence targets, finding ways to mitigate the LPE may have real-world consequences. Here we demonstrate that interspersing “probe” trials—trials with known targets and post-response feedback—reliably reduces the LPE. We also investigate the extent to which the probe targets must match the real targets and use eye tracking to determine the search mechanism impacted by probes. Our results suggest that the probe benefit is derived from increasing top-down guidance toward items that share features with frequently probed targets, suggesting that to be effective, probe targets would need to share features with targets in the real search task. Finally, we investigate whether the probe benefit results from the feedback they provide or because their inclusion increases the perceived prevalence rate of targets. Results suggest that the probe benefit results from a change in the perceived prevalence rate of targets. These findings may have implications for improving the detection of rare targets in real-world tasks and provide some insight into the types of probes that would be required for such an approach to be successful.

## Introduction

While visual search tasks have been ubiquitous in attentional research, most of that work had targets present on 50% or 100% of trials. While research in radiology first raised the concern that target prevalence rates might impact search performance (Horowitz, 2017; Kundel, 1982), Wolfe and colleagues (Wolfe et al., 2005) were the first visual cognition researchers to explore this issue. They found that as targets in a task become rare, the likelihood of missing them (when they do occur) skyrockets, an effect known as the low-prevalence effect (LPE). The LPE is concerning because it may have implications for a number of important real-world tasks, such as mammography and baggage screening, in which target prevalence rates can be extremely low (Gur et al., 2004; Horowitz, 2017).

This worry has been justified based on findings suggesting that the LPE may influence target detection rates in real-world scenarios. For instance, trained radiologists (Evans et al., 2011) and TSA agents (Wolfe et al., 2013) are susceptible to the LPE, and drivers are less likely to notice road hazards when they become rare (Kosovicheva et al., 2023; Song & Wolfe, 2024). These potential real-world consequences have driven research investigating potential methods to reduce the LPE and improve low-prevalence search.

However, much of this research has found that the LPE is particularly stubborn and difficult to mitigate (Van Wert et al., 2006; Wolfe et al., 2007). For instance, to reduce miss errors researchers have attempted to compel observers to perform a more complete search prior to executing a target absent response. One method of doing so was by using an eye tracker to provide participants with real-time feedback about which sections of the search display had not yet been fixated. This type of real-time feedback did not help; it did not result in a more thorough search of the displays, nor did it impact the magnitude of the LPE (Drew & Williams, 2017; Peltier & Becker, 2017). While that result may be surprising, it is only one of a number of attempts to improve low-prevalence search that have failed to reduce the LPE. For instance, the LPE persists even when the search is shifted to a feature search (Rich et al., 2008); video recording participants during their search improves overall detection but does not reduce the LPE (Miyazaki, 2015); splitting displays so that only half the items appear in each of the two epochs fails to reduce the LPE (Kunar et al., 2010); and implementing a delay before people can respond fails to eliminate the LPE (Wolfe et al., 2007). Having two people perform the search (known as “double reading”) is less conclusive, with some showing it reduces the LPE (Kunar et al., 2021) and others showing it does not (Wolfe et al., 2007). However, even if it does, trying to implement double reading in a real-world scenario may be prohibitively expensive.

By contrast, one recent method has been shown to eliminate the LPE. Taylor and colleagues (Taylor et al., 2022) altered the task instructions—changing from a target present/absent response to identifying which item in the search array *most resembled* the target. They found that this change in instructions completely eliminated the LPE, and in target present trials the selected item was frequently the actual target. While an interesting finding, from a practical perspective this change in instructions would be problematic, as it results in selection akin to a false alarm in every trial without a target—the vast number of trials in a low-prevalence search. Or to put it another way, you would not want to be in a busy TSA screening line that utilized this approach. Gillies and Kosovicheva (2025) noticed this potential flaw and instructed participants to perform a two-stage task. In the first stage, participants selected the item that was most similar to the target, and in the second stage, they made a binary decision about whether the selected item was actually a target or not. Unfortunately, the LPE returned for this second binary decision, limiting the real-world viability of such an approach.

However, Wolfe and colleagues discovered an alternative approach that successfully reduces the LPE and appears to have real-world feasibility (Wolfe et al., 2007). Their approach (Experiment 7) was to insert an additional mini-block of trials into the middle of the LPE search task. The mini-block involved a set of high-prevalence trials (50% prevalence rate) that involved post-response feedback; during these trials, the search array remained on the screen after the participant’s response and the target was circled in red with text either praising the participant for finding the target (when correct) or telling them they had missed the target. While feedback was given during this high-prevalence mini-block, targets were rare and feedback was absent in the other “true” trials. Even so, they showed that including the mini-block reduced the LPE. In theory, this approach could be implemented in real-world scenarios. For instance, in mammography it would amount to inserting a set of scans with known cancers into the middle of the radiologist’s workload and providing feedback after each of those known scans was evaluated.

Given the potential success of this method, we first sought to replicate their findings using a slightly modified method of presenting the additional “probe” trials. We reasoned that rather than including them in a mini-block, dispersing a set of “probe” target present trials throughout the task may also be effective. If so, it might confer some additional real-world advantages, because there would not be a prolonged period when the observer was off task, and it might allow agencies to track an individual’s performance over time to identify when performance dips and a break is needed.

Indeed, this approach is similar to the Threat Image Projection (TIP) system that many countries mandate as part of airline baggage screening (Catchpole et al., 2023). The TIP system projects a fictitious threat image (e.g., gun, explosive) into travelers’ bags as they are screened through the X-ray machines at security checkpoints. When the security screener detects the threat, they are provided with immediate feedback that the image was a TIP image. This system is used during training and to monitor on-the-job performance of individual operators. If an operator’s monthly performance drops below a given standard, the operator must attended remedial training (U.S. Government Accountability Office, 2016). To date, the primary use of the program is to enhance training and monitor performance. However, if we find that these types of probes increase rare-target detection, it is possible that the use of the program has a direct benefit for rare-target detection (Cutler & Paddock, 2009), rather than just a training benefit.

To foreshadow, Experiment 1 investigates this dispersed probe method, Experiment 2 then investigates whether the probe benefit generalizes to targets whose features do not match the probe, Experiment 3 uses eye tracking to attempt to determine the mechanism responsible for the probe benefit, and Experiment 4 investigates whether the feedback provided in probe trials is essential for their benefit or whether they provide benefit because they change the perceived prevalence of specific targets.

## Experiment 1

The main goal of our first experiment was to replicate Wolfe et al.’s (2007) mini-block advantage and determine whether dispersing the mini-block’s “probe trials” throughout the trial sequence would confer a similar advantage.

### Participants

A power analysis calculating the sample size required to evaluate the interaction term of a mixed-model ANOVA with an effect size of.18 (halfway between a small and moderate effect size) with power of.95, suggested an overall sample size of 104. Assuming some participants would be non-compliant, we ran a total of 111 participants. Data from nine participants were eliminated from further analyses due to false alarm rates > 45%, leaving a final sample of 102 participants. False alarm rates for the remaining participants were extremely low (*M* = 1.1%, *SE* =.45%). All participants were undergraduate students recruited through our campus SONA system and participated for course credit or extra credit. All participants reported normal or correct to normal vision. All methods were approved by Michigan State University’s IRB and participants gave informed consent. We did not ask for age or gender information.

### Procedure

The experiment was programed in E-Prime and ran in sound attenuated testing rooms on PCs with 24-inch monitors set at a resolution of 1024 by 768 running at 60 Hz. Each participant completed a control block and an experimental block, with the block order randomized. For roughly half (*n* = 52) the participants the experimental block included a “mini-block” of trials with feedback similar to Wolfe’s approach. For the other participants (*n* = 50), the experimental condition included “probe trials” which interleaved a set of target present trials with feedback throughout the block with the “real” trials. Each condition began with 50 practice trials with 10% target prevalence and no feedback to allow observers to set prevalence-appropriate quitting thresholds and decision criterion for a low-prevalence task (Ishibashi et al., 2012). The control block consisted of 250 trials with a 10% prevalence rate and no feedback. Both experimental blocks consisted of 250 “real” trials with a 10% prevalence rate and no feedback plus an additional 50 trials with feedback. The mini-block intervention involved a set of 50 trials with 50% prevalence rate and feedback that was presented halfway through the block of real trials. The probe intervention involved 50 target present trials with feedback that were randomly dispersed throughout the real trials.

**Fig. 1:**
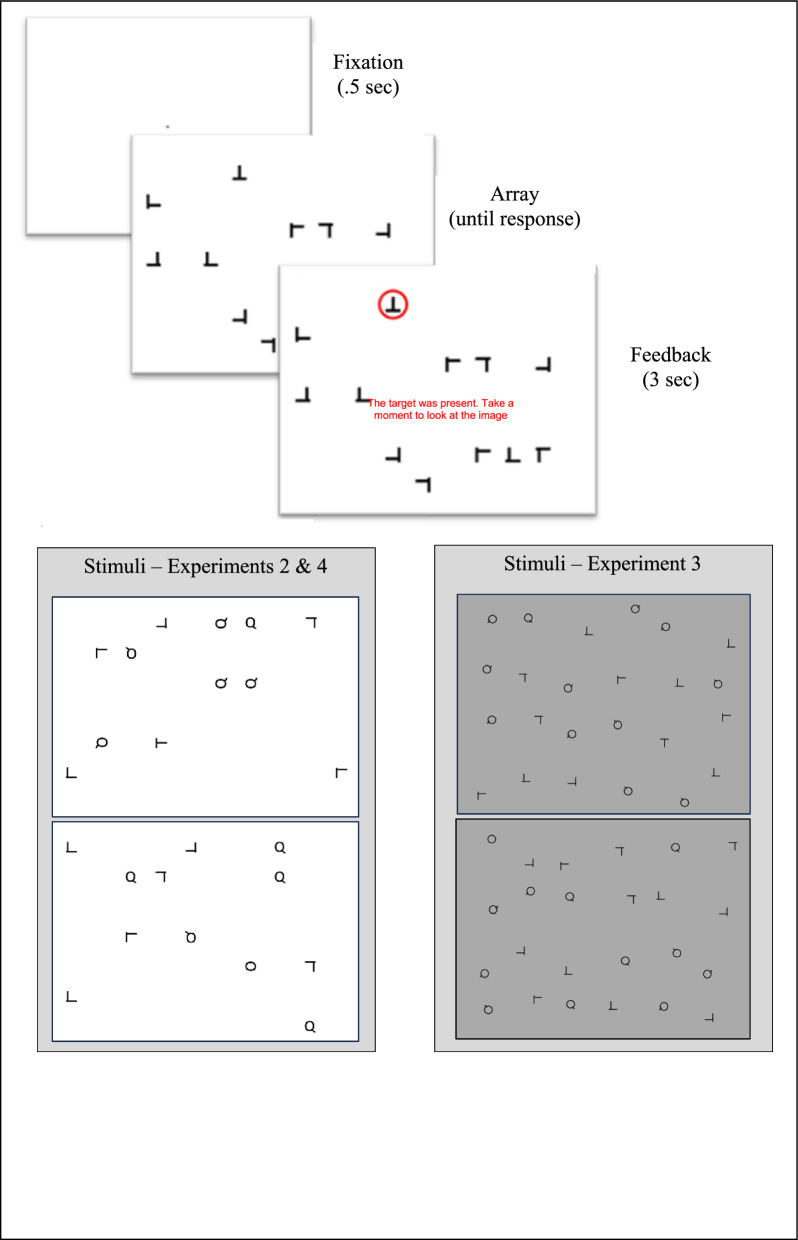
The top depicts an example of a target present trial with feedback from Experiment 1. Note that the feedback depicted only occurred for probe intervention trials. In “real” trials, the next trial’s fixation screen would directly follow the response. The lower panels depict examples of target present stimuli from later experiments. Top examples have a T target and lower examples have a O target. All experiments followed the same trials structure as Experiment 1

Each trial began with a central fixation cross (.5 s), followed by the search array (see Fig. 1). For real trials, the array remained on the screen until the participants made a target present/target absent button response, and then the next trial would begin with its fixation cross. All probe and mini-block trials were followed by feedback. In the probe and mini-block trials, the participant’s button press did not erase the array. If the trial was a target present trial (all probe trials and ½ the mini-block trials), a red circle would appear around the target and the words “The target was present. Take a moment to look at the image” would appear in the middle of the screen. If the trial was a target absent trial (only in mini-block condition), the feedback consisted of the words “The target was absent. Take a moment to look at the image” appearing in the center of the screen. The feedback would be displayed for 3 s, and then the next trial would begin with its fixation cross.

#### Displays

Target absent displays consisted of 12 black items on a white background. Each was an offset L (for the vertical presentation: ~ 2.3° × ~ 2° with the vertical component indented by ~.3° from the edge of the horizontal line, and a line width of ~.25°) randomly presented at any of the four cardinal directions. In target present displays, one of the offset Ls was replaced by a T appearing in any of the cardinal directions. The screen (~ 52° × 29.5°) was segmented into 70 possible locations (10 × 7 grid) and array items appeared in a randomly selected set of 12 locations.

### Results

All analyses were performed only on the non-probe, “real” trials. A series of 2 × 2 mixed-model ANOVAs with Block (Control/Experimental) as a within-factor and Intervention Type (Mini-Block/Probe) as a between-factor were performed, with hit rate, false alarm rate, hit reaction time (RT), and target absent RT as dependent variables in separate analyses.

#### Accuracy

**Fig. 2:**
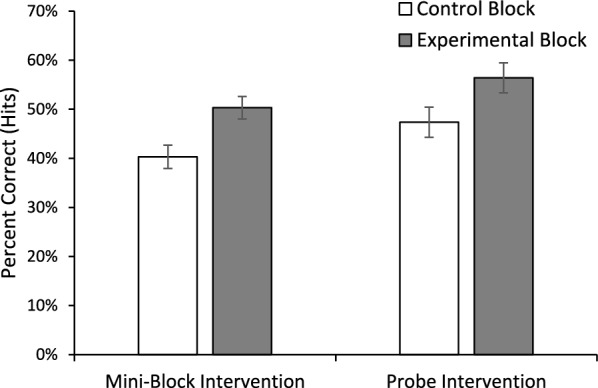
Mean hit accuracy for control and intervention blocks as a function of which intervention the subject received. Error bars are the standard error of the mean

For hits, there was main effect of Block, *F*(1, 100) = 32.928, *p* <.001, *η*_*p*_^2^ =.248, with higher accuracy for the experimental blocks than the control blocks (see Fig. 2). The main effect of Intervention Type was not significant, *F*(1, 100) = 3.584, *p* =.061, *η*_*p*_^2^ =.035, but there was a trend for overall better performance in the probe intervention than the mini-block intervention. This trend may be due to the fact there were more target present trials in the probe intervention, since 100% of the probes had targets, while only 50% of the mini-block trials had targets. The two factors did not interact, *F*(1, 100) =.085, *p* =.772, *η*_*p*_^2^ =.001. False alarms were rare; all conditions had a false alarm rate < 2.2%. Neither main effect nor the interaction approached significance, all *F*(1, 100) < 1, all *p* >.35, all *η*_*p*_^2^ <.01. Thus, the higher accuracy in the experimental conditions can be attributed to a shift in sensitivity rather than a change in criterion.^1^

#### Reaction time

**Fig. 3:**
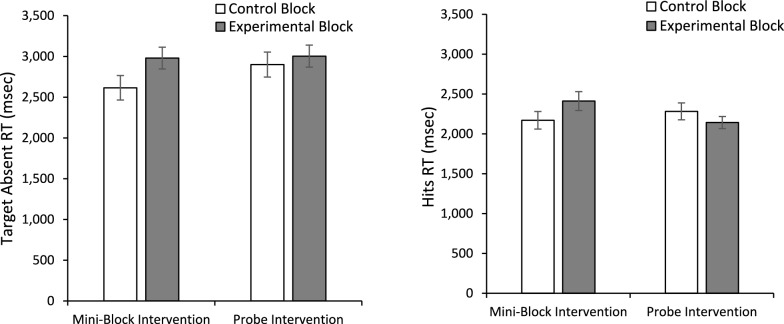
Mean reaction time for correct rejections (left panel) and hits (right panel) as a function of experimental block and the type of intervention the subject received. Error bars are the standard error of the mean

One explanation that has been offered for the low-prevalence effect is that low prevalence causes a shift to a lower quitting threshold (Peltier & Becker, 2016; Wolfe & Van Wert, 2010; Wolfe et al., 2005); people search less of the display before responding target absent, thereby increasing the miss rate and producing faster target absent responses. Thus, one possibility is that the experimental interventions improve performance because they shift the quitting threshold higher. To examine this possibility, we performed an ANOVA on target absent RTs (Fig. 3). Consistent with this view, there was main effect of Block, *F*(1, 100) = 8.416, *p* =.005, *η*_*p*_^2^ =.078, with faster target absent RTs in the Control Block (*M* = 2757.29, *SE* = 107.13) than the Experimental Blocks (*M* = 2991.36, *SE* = 95.01). There was no main effect of Intervention Type, *F*(1, 100) =.691, *p* =.408, *η*_*p*_^2^ =.007, nor an interaction, *F*(1, 100) = 2.628, *p* =.108, *η*_*p*_^2^ =.026.

For completeness we also performed an ANOVA on hit RTs (Fig. 3). Neither main effect approached significance, both *F*(1, 100) < 1, both *p* >.5, both *η*_*p*_^2^ <.005. However, there was a significant interaction, *F*(1, 100) = 6.305, *p* =.014, *η*_*p*_^2^ =.059. The source of the interaction appears to be that the intervention produced slower hit RTs than control in the mini-block condition, but faster RTs than control in the probe condition, although Bonferroni correct follow-up comparison reveals this difference was only significant for the mini-block Intervention (*p* =.025) and did reach significance for the probe condition (*p* =.20).

### Discussion

Replicating Wolfe et al. (2007), we found that including a high-prevalence mini-block of trials that provide post-response feedback within an otherwise low-prevalence search task improved rare-target detection. In addition, our results suggest that distributing the probe trials throughout the block, rather than presenting them in a mini-block, is equally effective at increasing rare-target detections. Further, we found that both interventions produced slower target absent RTs than in the control block. This change in target absent RTs is consistent with the interventions increasing quitting thresholds, thereby leading to longer and more thorough searches before responding target absent.

Since it appears that both the probe and mini-block methods produce similar benefits, going forward we will test only the distributed probe method developed here. We do so because we believe the probe technique might have some practical advantages—distributed probes could allow one to monitor a searcher’s performance over time and the intervention involves a brief single trial at a time, rather than a prolonged mini-block of trials.

Given the success of these interventions in improving rare-target search, their implementation in real-world search contexts, such as TSA screening or radiology, might provide real-world benefit. However, before making such a suggestion, it is worth noting that in our experiments participants were searching for a single, well-defined target—in our case a letter “T”—and the probe trial targets perfectly matched the targets in the real trials. This raises the question of how closely the targets during probe trials have to match the actual targets to achieve a benefit. From a practical stance, a probe benefit that generalizes beyond targets that closely match the probed targets would be valuable.

The original Wolfe et al. (2007) experiment using mini-blocks provides some insight into this issue. The task required people to search for knives and guns; multiple exemplars of both types of targets were used, and the probe targets were not the exemplars used in the real trials. Thus, their data suggest that probes do not have to be an exact match to the real targets to result in a benefit. Still, it is likely that there was substantial overlap in the features of the probe targets and the real targets in their experiment. They also had 50% of their probe targets match each target category, so each target type was moderately probed. What would be more informative is to have probes match one category more frequently than the other and determine whether the probe benefit generalized to an infrequently probed target. This approach would provide more insight into how well probe benefits generalize to non-probed targets and might provide insight into the mechanisms responsible for the probe benefit.

Three mechanisms have been associated with miss errors in low-prevalence search scenarios. One is the trial-wide quitting threshold—a factor that determines how completely the search array is inspected before making a target absent response (Chun & Wolfe, 1996; Peltier & Becker, 2016; Wolfe & Van Wert, 2010). If targets become rare, the number of items inspected prior to making a target absent response may decrease, leading to more misses and faster target absent RTs (Peltier & Becker, 2016; Wolfe et al., 2005). If probes simply increase this trial-wide quitting threshold, a more thorough search may benefit all targets—even those that do not match probed targets. A second potential mechanism that could impact miss rates is the criterion for identification of a target when evaluating whether a fixated item is a target or not (Hout et al., 2015; Peltier & Becker, 2016; Wolfe & Van Wert, 2010). If probes make this criterion more liberal for the probed target, then the probe benefit might be selective to targets that match the probes (and there might be more false alarms identifying distractors as this type of target). Finally, it is possible that probes increase top-down guidance (Chen & Zelinsky, 2006) toward items that share the frequently probed targets’ features (Hout et al., 2014). If this were the case, the probe benefit would be selective to targets that matched the probed target. Of course, it is also possible that the probe benefit impacts more than one of these factors. For instance, if an increase in the quitting threshold co-occurs with more top-down guidance to the features associated with the frequently probed target, the benefit may still be confined primarily to targets that match or closely match the targets that appear during the probe trials. To investigate this issue in Experiment 2, we had subjects search for two very distinct targets and varied the proportion of probe trials that matched each.

## Experiment 2

To investigate whether the probe benefit relies on the probed targets being similar to the actual targets, we ran a series of experiments in which subjects simultaneously searched for either of two targets—a “T” or “O”—among arrays of 12 items. On target absent trials, half the items were offset L distractors and half were Q distractors, providing a set of distractors that was visually similar to one of the targets (T and Ls; O and Qs) and dissimilar to the other (T and Qs; O and Ls). Each participant completed two blocks of trials—a control block and a probe block—with the order of presentation counterbalanced across subjects. The T and L stimuli were identical to Experiment 1; the O was an oval (~ 2° × ~ 2.3°) and the Q was the same oval with a “tail” attached to the oval (~.6°). Each experiment began with 40 practice trials, with 10% prevalence rates for each target. In the control block of 150 real trials, each target appeared on 15 trials for a 10% prevalence rate for each target. When the O target appeared, it replaced a Q distractor in the display, and when a T target appeared it replaced an L distractor, resulting in every display having six O-like stimuli and six T-like stimuli. Target present trials never had both targets present. After the initial practice trials, the probe block consisted of the same 150 trials as the control block plus an additional 50 probe trials that were randomly distributed throughout the block. These probe trials were target present trials that presented the same post-response feedback as in Experiment 1. No feedback was provided in non-probe, real trials. There were three versions of the probe block that varied how frequently the targets in the probe trials matched one of the targets versus the other. In one version, 100% of the probes matched one target, with none matching the other target. In a second version, one target appeared in 80% of the probe trials, with the other target appearing in the remaining 20%. In a final version, both targets appeared in 50% of the probe trials. To avoid confusion, we will refer to this manipulation of the relative frequency of probes as “probe balance.” In all versions, which target was assigned to each probe prevalence condition was counterbalanced across participants. Participants responded in the same way as Experiment 1, they pressed one button when they detected either target or a second button to indicate target absent.

### Participants

A total of 172 participants were recruited. Data from two were eliminated due to a computer crash before completing the second block of trials. Data from an additional seven were eliminated for false alarm rates > 45%, and data from three additional subjects were eliminated for hit rates less than 3 standard deviations from the mean hit rate, leaving a final sample size of 160 participants, with 67 participants in the 100/0 balance condition, 28 participants in the 50/50 balance condition, and 65 participants in the 80/20 balance condition. We had fewer subjects participate in the 50/50 condition because each target was probed at the same rate, so data were pooled across targets yielding twice as many trials per subject in the analyses. Like Experiment 1, all subjects participated for course credit through our SONA system. All subjects reported normal or correct to normal vision.

### Results

**Fig. 4:**
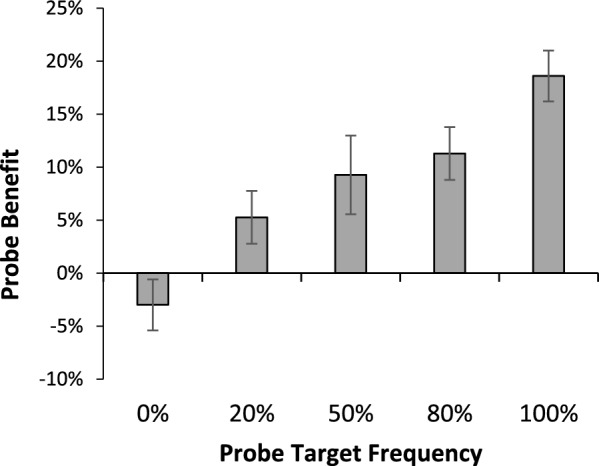
Mean probe benefit (probe block hit rate—control block hit rate) for each target as a function of how often that target appeared as target in the probe trials. Error bars depict the standard error of the mean

Accuracy data were calculated only on the non-probe trials. For ease of comparisons for each probe prevalence rate, we calculated the magnitude of the probe benefit, defined as the difference in hit rate between probe and control blocks. A between-subjects ANOVA with five levels of probe prevalence (0%, 20%, 50%, 80%, 100%) was conducted on these probe benefit scores (Fig. 4). There was a main effect of probe frequency, *F*(4, 281) = 10.881, *p* <.001, *η*^2^ =.134. Pairwise follow-up comparisons showed that the 0% probe benefit was significantly lower than all other probe benefits, all *p* <.018, and the 100% probe benefit was significantly greater than all other probe benefits, all *p* <.037. We also performed single sample t tests for each probe prevalence rate to determine whether the probe benefit was significantly greater than zero. These analyses showed no probe benefit for the target that never appeared during the probe trials,* t*(66) = −1.326, *p* =.19, *d* = -.162. However, all other probe frequencies showed a significant positive probe benefit [20% *t*(61) = 2.004, *p* =.05, *d* =.254; 50% *t*(27) = 3.708, *p* <.001, *d* =.701; 80% *t*(61) = 4.758, *p* <.001, *d* =.604; 100% *t*(66) = 6.728, *p* <.001, *d* =.822].

False alarms were extremely rare with all conditions having a mean false alarm less than 1%. Given this ceiling performance, it is not surprising that a mixed-model ANOVA with Block (Control/Probe) as a within factor and Probe Balance Condition (0/100, 50/50, 80/20) as a between factor found no main effect of Block, Probe Balance Condition, or interaction, all *F* < 1.14, all *p* >.32, all *η*_*p*_^2^ <.015. Note there is no probe prevalence factor in this analysis because it is based on target absent trials.

### Reaction time

**Fig. 5:**
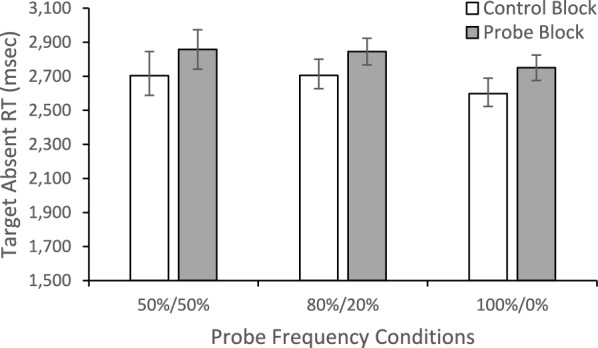
Reaction times for correct rejections as a function of probe frequency conditions and experimental block. Error bars depict the standard error of the mean

We again looked for evidence that the probes were increasing quitting thresholds. To do so we performed a mixed measures ANOVA on target absent RTs with Block (Control/Probe) as a within-subjects factor and Probe Balance Condition (100/0, 50/50, 80/20) as a between-subjects factor (Fig. 5). There was main effect of Block, *F*(1, 154) = 8.195, *p* =.005, *η*_*p*_^2^ =.051, with faster target absent RTs in the control (*M* = 2669.14, *SE* = 64.43) than the probe blocks (*M* = 2817.78, *SE* = 52.80). Neither the main effect of Probe Balance Condition nor the interaction approached significance, both *F* < 1, both *η*_*p*_^2^ <.009.

To investigate how probes and their frequency impacted target present RTs, we began with an omnibus mixed-model ANOVA with Block (Control/Probe) and Probe Prevalence Rate (High/Low) as within-subjects factors and Probe Balance Condition (100/0, 80/20) as a between-subjects factor. Note that we dropped the 50/50 Probe Balance Condition from this analysis because there was not a high/low-prevalence rate for the two targets as each was probed at 50%. There was a main effect of Probe Prevalence Rate, *F*(1, 127) = 17.997, *p* <.001, *η*_*p*_^2^ =.124, with faster RTs for detecting frequently probed targets (*M* = 2029.48, *SE* = 40.84) than less frequently probed targets (*M* = 2181.31, *SE* = 35.59). Prevalence rate also interacted with the Probe Balance Condition, *F*(1,127) = 4.98, *p* =.027, *η*_*p*_^2^ =.038, and Block, *F*(1, 127) = 14.348, *p* <.001 *η*_*p*_^2^ =.102. There was not a main effect of Block, *F* < 1, *η*_*p*_^2^ <.001, and Block did not interact with Probe Balance Condition, *F*(1, 127) = 1.658, *p* =.20, *η*_*p*_^2^ =.013. The main effect of Probe Balance was not significant, *F*(1, 127) = 2.254, *p* =.136, *η*_*p*_^2^ =.017, nor was the three-way interaction, *F* < 1, *η*_*p*_^2^ =.001.

**Fig. 6:**
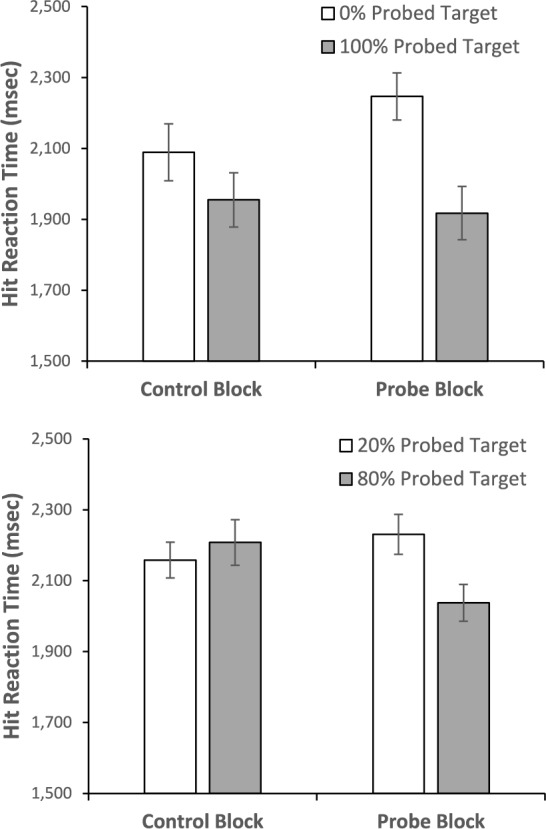
Hit reaction times for targets that matched the frequently or less frequently probed targets as function of experimental block. The top panel presents results from the version of the experiment where probes matched one of the targets 100% of the time. The lower panel is for the 80% and 20% version. Error bars represent the SEM

To unpack the interactions, we ran two 2 (Block: Control/Probe) × 2 (Prevalence Rate: High/Low) within-subjects ANOVAs (Fig. 6), one for each of the Probe Balance Conditions (100/0% and 80/20%). For the 100/0% Probe Balance Condition, the main effect of Block was not significant, *F*(1, 66) =.814, *p* =.37, *η*_*p*_^2^ =.012. However, there was a main effect of Prevalence Rate, *F*(1, 66) = 17.77, *p* <.001, *η*_*p*_^2^ =.212, and a Prevalence Rate by Block interaction, *F*(1, 66) = 4.045, *p* =.048, *η*_*p*_^2^ =.058. The source of the interaction is that there was no significant difference, *F*(1, 66) = 3.727, *p* =.058, *η*_*p*_^2^ =.053, between the high probed and low probed items in the control condition, but the target that matched the probed target was detected more quickly than the target that never matched the probed targets in the probe condition, *F*(1, 66) = 18.417, *p* <.001, *η*_*p*_^2^ =.218. For the 80/20% Probe Balance Condition, neither main effect was significant, both *F*(1, 61) < 2.58, both *p* >.11, both *η*_*p*_^2^ <.042. However, there was a significant Probe Frequency by Block interaction, *F*(1, 61) = 16.988, *p* <.001, *η*_*p*_^2^ =.218. Again this interaction resulted because there was no difference between the two targets in the control condition, *F*(1, 61) =.755, *p* =.388, *η*_*p*_^2^ =.012, but in the probe condition, the target that was probed 80% was detected more rapidly than the target that was probed 20%, *F*(1, 61) = 14.847, *p* <.001, *η*_*p*_^2^ =.196.

### Discussion

Across all versions we found evidence that the probe technique can increase target detection rates for rare targets, even when participants searched for two unique targets. In addition, those increased target detection rates occurred without changing false alarm rates, suggesting that the probes are increasing discrimination of targets rather than shifting criterion (see footnote 1). However, it is also clear from these data that the magnitude of the probe benefit seems to scale with how frequently the target in the probe trials matches a particular target in the real trials. Although this relationship is clear from the linear trend in Fig. 4, the most telling data come from the version where 100% of the probes matched one of the search targets, while 0% matched the other. In that version, the 100% probed target experienced the largest probe benefit of all probe frequency conditions. Moreover, the 0% probed target showed absolutely no probe benefit. Thus, it appears that the ability of a probe to benefit target detection relies critically on the targets in the probe trials being similar to the targets in real trials.

We also found that the target absent RTs were longer in the probe blocks than control blocks. This finding is consistent with the view that the probes improve target detection because they increase the trial-wide quitting threshold (Peltier & Becker, 2016; Wolfe & Van Wert, 2010; Wolfe et al., 2005). The higher quitting threshold results in a more thorough search of the display before responding target absent, thereby increasing hits and slowing target absent RTs. In theory, increasing the quitting threshold might have produced a benefit that extended to targets that did not match the probed targets—if the probes make search more thorough, they might have benefitted any target. However, we do not find that the probe benefit generalized to targets that did not appear in the probe trials.

One possible explanation for this failure of generalization is that the probes might have produced both an increase in quitting thresholds and an increase in top-down bias toward features associated with the frequently probed targets. Although one might have searched more items in the probe block, the bias to search items with features matching the probed targets might have limited the benefit of a more thorough search to targets with features associated with the frequently probed targets.

To more fully investigate these potential mechanisms, Experiment 3 included eye tracking, which allows for a more detailed analysis of how probes influence different search mechanisms.

## Experiment 3

Experiment 3 involved replicating the 80/20% probe balance version of Experiment 2 while tracking eye movements. The eye movement data should allow us to independently evaluate the contribution of the three search mechanisms that the probes may be impacting. If the probes increase quitting thresholds, we would expect that more unique items to be fixated (scrutinized) prior to executing a target absent response. If probes increase top-down guidance toward frequently probed features, we would expect more fixations to be made on distractors that shared features with the frequently probed target (e.g., Ls when Ts where frequently probed) than distractors that shared features with the infrequently probed target (e.g. Ls when Os were frequently probed). We would also expect more direct guidance to the frequently probed target, resulting in faster target present RTs and fixations to fewer unique items before making a hit. In addition, this top-down bias would predict more search errors, miss errors where the target was never fixated, for infrequently probed than frequently probed targets. If probes resulted in a more liberal item-by-item decision criterion for identifying an object as the frequently probed item, we would expect fewer identification errors (miss errors where the target was fixated but still not identified) for targets matching the highly probed target.

### Methods

#### Procedure

The method was similar to the version of Experiment 2 that had 80/20% probe balance. The main differences were that participants performed the experiment, while their eye movements were monitored by an EyeLink 1000 eye tracker, with monocular sampling of eye positions at 1000 Hz. We also increased the set size from 12 items to 24, to encourage more eye movements, and we presented the stimuli as black items on a light gray background to eliminate glare from the screen (see Fig. 1). The O stimulus was also rendered as a circle (diameter 1.9°) rather than an oval, and the Q was the same circle with a “tail” (.6°). To implement eye tracking, participants used a headrest located 57.2 cm from the screen and performed the EyeLink’s standard nine-point calibration and verification technique prior to beginning the experiment. The raw eye-position data were parsed into fixations using the EyeLink DataViewer’s parser with the default parameters. Finally, there was a fixation point used to verify accurate eye tracking between each trial. If this drift check found that the eye was no longer being accurately tracked, the calibration and verification process was run again prior to the next trial.

#### Participants

A power analysis for a within-subjects ANOVA with four measures, a medium effect size of *f* =.25 (*η*_*p*_^2^ ~.06), and power =.90 suggested a sample size of 30 would be adequate. We chose that effect size because it is the conventional effect size for a medium-sized effect, most of our prior effect sizes were medium to large, but we felt it is inappropriate to use those actual effect sizes because the dependent variables of interest in this experiment were primarily eye tracking measures that were not included in prior experiments. A new set of 33 participants were recruited for Experiment 3. One subject was eliminated for false alarm rates >.45, leaving a final sample size of 32 participants. Like prior experiments, all were recruited via our SONA system, were undergraduates participating for course credit or extra credit, gave informed consent, and reported normal or corrected to normal vision.

### Results

#### Accuracy

**Fig. 7:**
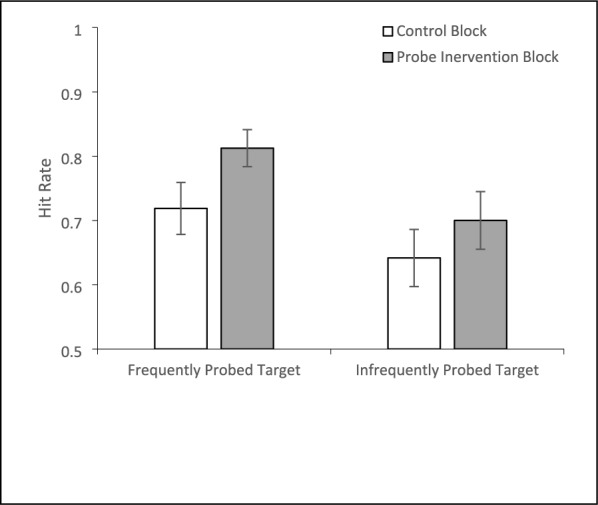
Hit accuracy for Experiment 3 as a function of block and the frequency of the target appearing in probe trials. Error bars depict the standard error of the mean

A 2 (Block: Control/Probe) × 2 (Probe Prevalence Rate: Low/High) repeated-measures ANOVA on hit rate (Fig. 7) found a significant main effect of Block, *F*(1, 31) = 12.396, *p* <.001, *η*_*p*_^2^ =.286, with higher accuracy in the probe block (*M* = 75.6%, *SE* = 2.7%) than the control block (*M* = 68.0%, *SE* = 3.3%). The main effect of probe frequency was marginally significant, F(1, 31) = 3.646, *p* =.065, *η*_*p*_^2^ =.105, with higher accuracy for the frequently probe target (*M* = 76.6%, *SE* = 3.2%) than the infrequently probed target (*M* = 67.1%, *SE* = 4.3%). The interaction was not significant, *F*(1, 31) = 1.053, *p* =.313, *η*_*p*_^2^ =.033. Planned paired t tests revealed a significantly higher hit accuracy during the probe block than the control block for both the highly probed, *t*(31) = 3.36, *p* =.002, *d* =.594, and less frequently probed targets, *t*(31) = 2.13, *p* =.041, *d* =.376. There was no significant difference between the high and low probed items in the control block, *t*(31) = 1.45, *p* =.16, *d* =.257, but the highly probed target outperformed the low probed target in the probe intervention block, *t*(31) = 2.16, *p* =.039, *d* =.382. A paired *t* test comparing false alarm rates in the control and probe block was insignificant, *t*(31) =.79, *p* =.435, *d* =.14.

#### Reaction times

**Fig. 8:**
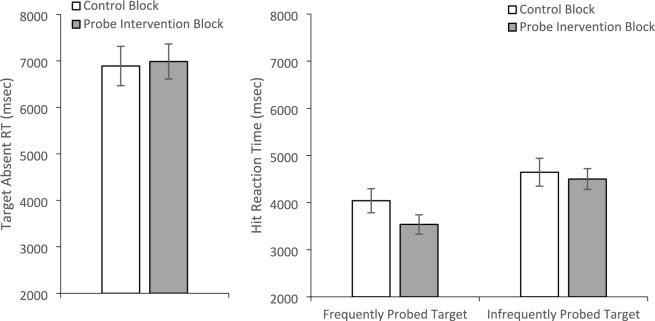
Reaction times for correct rejections (left panel) and Hits (right panel) as a function of Block and, for Hits, the frequency of the target appearing in probe trials. Error bars depict the standard error of the mean

Unlike prior experiments, a paired t test comparing target absent RTs (Fig. 8) between the probe (*M* = 6889.75, *SE* = 423.14) and control (*M* = 6986.21, *SE* = 375.48) block was insignificant, *t*(31) =.448, *p* =.66, *d* =.079. Given that we usually find this effect, we also performed a Bayesian paired-samples t test to compare target absent response times between the control and probe target absent RTs. The Bayes factor in favor of the null hypothesis over the alternative was BF_01_ = 6.62, indicating substantial evidence for the null hypothesis (Jeffreys, 1961). A 2 (Block: Control/Probe) × 2 (Probe Prevalence Rate: Low/High) ANOVA on the hit RTs (Fig. 8) found a main effect of Block, *F*(1, 29) = 8.04, *p* =.008, *η*_*p*_^2^ =.217, with faster RTs in the probe block (*M* = 4008.746, *SE* = 180.035) than the control block (*M* = 4349.533, *SE* = 218.126). There was also a main effect of probe frequency, *F*(1, 29) = 12.19, *p* =.002, *η*_*p*_^2^ =.296, with faster RTs for the highly probed target (*M* = 3783.864, *SE* = 210.214) than the less frequently probed target (*M* = 4574.415, *SE* = 232.841). While the interaction between the two factors did not reach significance, *F*(1, 29) = 2.788, *p* =.106, *η*_*p*_^2^ =.088, planned paired t test revealed that RTs were faster in the probe block than the control block for the highly probed item, *t*(30) = 3.795, *p* <.001, *d* =.682, but not for the infrequently probed item, *t*(30) =.483, *p* =.632, *d* =.087.

### Eye movement analyses

#### Number of unique items fixated

As a more direct measure of a change in quitting threshold, we calculated the number of unique items fixated in each target absent trial. If probes increased quitting thresholds, we would expect more unique items to be fixated prior to making a correct target absent response in the probe than the control block. We also limited this analysis to target absent trials to avoid the influence of finding the target in the analysis. A paired t test comparing the number of unique items fixated in the control (*M* = 16.47, *SE* =.65) and probe (*M* = 16.77, *SE* =.65) blocks found no significant difference, *t*(31) =.882, *p* =.384, *d* =.156.

#### Types of errors

**Fig. 9:**
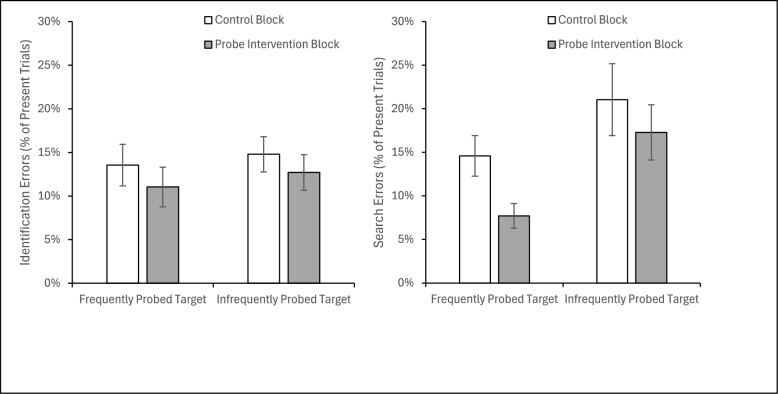
The percentage of target present trials that resulted in identification errors and search errors are plotted as a function of block and frequency of the target appearing during probe trials. Error bars depict the standard error of the mean

Misses were classified (Fig. 9) as either identification errors (misses where the target was fixated but still not identified as the target) or search errors (when the target was never fixated). To determine whether probes impact the item-by-item criterion used to evaluate whether an inspected item is a target or not, we analyzed the rate of identification errors. A 2 (Block: Control/Probe) × 2 (Probe Frequency: High/Low) within-subjects ANOVA on percentage of identification errors found that neither main effect nor the interaction was significant, all *F*(1, 31) < 1.95, all *p* >.17, all *η*_*p*_^2^ <.06. Thus, it appears probes do not impact the item-by-item identification criterion.

A similar 2 × 2 within-subject ANOVA on the percentage of search errors found a main effect of Block, *F*(1, 31) = 6.742, *p* =.014, *η*_*p*_^2^ =.179, with fewer search errors in the probe block (*M* = 12.5%, *SE* = 2.1%) than the control block (*M* = 17.8%, *SE* = 2.6%). There was also a main effect of probe frequency, *F*(1, 31) = 6.728, *p* =.014, *η*_*p*_^2^ =.178, with fewer search errors for the highly probed target (*M* = 11.1%, *SE* = 1.5%) than the infrequently probed target (*M* = 19.2, *SE* = 3.4%). Although the interaction was not significant, *F*(1, 31) = 1.069, *p* =.309, *η*_*p*_^2^ =.033, planned paired t test reveals that there were fewer search errors in the probe than control block for the highly probed target, *t*(31) = 2.953, *p* =.006, *d* =.522, but not for the infrequently probed target, *t*(31) = 1.367, *p* =.181, *d* =.242. This pattern of findings suggests that there is increased top-down guidance to the frequently probed target.

**Fig. 10:**
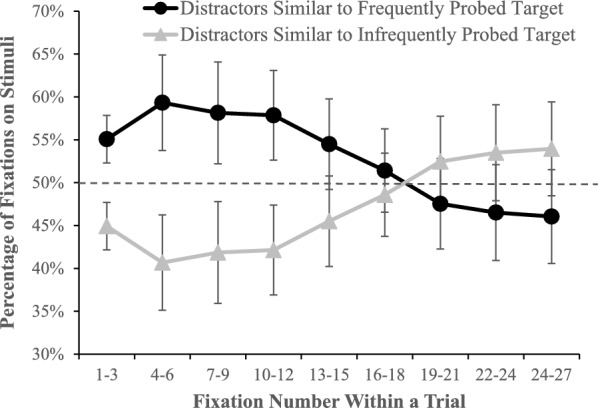
The percentage of fixations on distractors that were similar to the frequently and infrequently probed targets as a function of the ordinal fixation number within a trial. Error bars depict the 95% confidence intervals, and the dashed line is chance performance

To further investigate whether probes influenced top-down guidance, we classified each fixation on a distractor in the probe block as being on a distractor similar to the frequently probed target (e.g., fixating on a Q when the O target was highly probed) or infrequently probed target (e.g., fixating on a Q distractor when the T target was highly probed). We limited this analysis for target absent trials, so the fixations would not be influenced by the presence of the target and calculated the percentage of fixations within bins of three fixations that were on each type of distractor (see Fig. 10). As is clear from the figure there is an initial bias to fixate distractors that are similar to the frequently probed target, which diminishes after about the 12th fixation in the trial. A 9 (Fixation bin) × 2 (Probe Match: distractors matching the highly probed target/distractors matching the infrequently probed target) within-subjects ANOVA revealed a marginal main effect of probe match, *F*(1, 31) = 4.09, *p* =.052, *η*_*p*_^2^ =.12, that was qualified by an interaction with Bin, *F*(8, 248) = 5.59, *p* <.001, *η*_*p*_^2^ =.153. Pair-wise comparisons reveal that the source of the interaction is that there was a bias to fixate distractors that were similar to the frequently probed target that was significant for the first four bins (up to the 12th fixation), all *F*(1, 31) > 7.8, all *p* <.01, all *η*_*p*_^2^ >.20. The fifth bin was marginally significant, *F*(1, 31) = 3.01, *p* =.09, *η*_*p*_^2^ =.09, and all other bins did not approach significance, all *F*(1, 31) < 2.17, all *p* >.15, all *η*_*p*_^2^ <.066. These data suggest an initial strong guidance toward features that match the frequently probed target. The fact that the influence of this bias diminishes over fixations could result from the bias becoming less impactful after one has unsuccessfully found the target after sampling a number of items or simply because the initial bias impacts the pool of remaining un-fixated items; as one fixates more distractors that match the frequently probed target, a higher proportion of the remaining items match the infrequently probed target. Regardless, the initial bias suggests that the presence of probes creates guidance toward features of the frequently probed target.

**Fig. 11:**
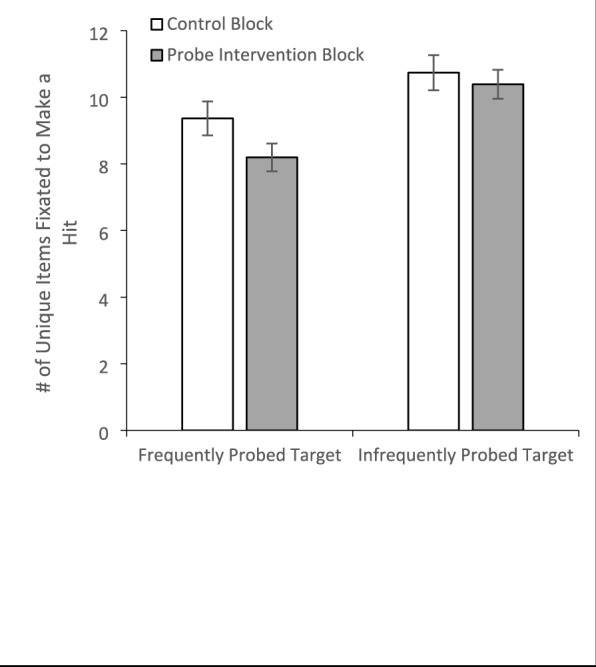
The mean number of unique items fixated in correct target present trials as a function of block and whether the target was the frequently probed or infrequently probed target. Error bars depict the standard error of the mean

As a final test of the theory that probes impact search guidance, we investigated the number of unique items fixated prior to making a correct target detection in a 2 (Block: Control/Probe) × 2 (Probe Frequency: High/Low) within-subjects ANOVA (Fig. 11). There was a main effect of Block, *F*(1, 29) = 5.83, *p* =.022, *η*_*p*_^2^ =.17, with fewer unique items fixated during the probe (*M* = 9.29, *SE* =.35) than the control block (*M* = 10.05, *SE* =.39). There was also a main effect of Probe Frequency, *F*(1, 29) = 11.19, *p* =.002, *η*_*p*_^2^ =.28, with fewer unique items fixated for the frequently probed target (*M* = 8.78, *SE* =.41) than the infrequently probed target (*M* = 10.56, *SE* =.45). While the interaction did not reach significance, *F*(1, 29) = 2.56, *p* =.12, *η*_*p*_^2^ =.08, planned comparisons found significantly fewer, *t*(30) = 2.60, *p* =.01, *d* =.47, fixations were required to find the highly probed target in the probe block than the control block. By contrast, there was no difference across blocks between the number of fixations required to find the infrequently probed target, *t*(30) =.33, *p* =.74, *d* =.06.

### Discussion

This eye tracking experiment replicated the finding from Experiments 1 and 2 that probes increased target detection rates, that this probe benefit was larger for the frequently probed target than an infrequently probed target, and that probes decreased target present RTs especially for targets that matched the frequently probed targets. In addition, the eye movement data showed that during target absent trials, there was a bias to fixate distractors that shared features with the frequently probed rather than the infrequently probed target. This finding suggests that probes increased top-down guidance toward their features (Hout et al., 2014). There was also evidence of this top-down bias in target present trials. Search errors, misses due to never fixating on the target, were less likely when the target matched the frequently probed than the infrequently probed target, suggesting a bias to look at items matching the frequently probed items. In short, one of the driving factors behind the probe benefit—and the reason it is selective to targets that share features with the frequently probed targets—is that probes increase top-down guidance to the features of the probed targets.

Unlike our earlier experiments, Experiment 3 failed to find that probes increased target absent reaction times. In addition, the eye movement data failed to find that more unique items were inspected in the probe than control conditions. If a main factor in the probe benefit was an increase in the trial-wide quitting threshold, we would have expected both of these effects to be significant, and thus this third experiment provides little evidence that changes in trial-wide quitting thresholds are a driving force in the probe benefit.

However, we would not want to completely rule out a role for changes in quitting thresholds in the probe benefit. The increase in target absent RTs in Experiments 1 and 2 provides consistent evidence that probes may increase quitting times. What we can conclude is that such an increase is not required to observe a probe benefit, because a substantial part of the benefit seems to be driven by top-down guidance toward features matching the probes. However, we cannot rule out that in some circumstances the probe benefit is also driven, at least somewhat, by changes in quitting thresholds.

There are a number of potential reasons why we might have failed to find evidence for a change in quitting thresholds in this experiment. First, it is possible that the addition of eye tracking produced demand characteristics to minimize misses, and these minimized any quitting threshold effects. Consistent, with this view, the overall accuracy rates in this experiment are far higher than in Experiments 1 and 2. In addition, in order to encourage more eye movements, we increased the set size from 12 to 24 items in the current experiment. This resulted in much longer RTs and a commensurate increase in variability around those RTs. This increase in variability may have made it more difficult to find a significant effect of probes on target absent RTs. In short, while the results from Experiment 3 provide strong evidence that top-down guidance plays a role in the probe benefit, the results from this experiment alone should not be taken as strong evidence against probes influencing overall quitting thresholds.

## Experiment 4

The results from Experiments 1–3 suggest that the use of probes is effective, the amount of benefit is related to the frequency with which the probed targets match the targets in the real trials, and the probes tend to increase guidance toward the features associated with frequently probed targets. Still, it is unclear whether the feedback provided during the probe trials is essential to their benefit. Alternatively, it might be that the inclusion of the probe trials simply changes the perceived prevalence rate for probed targets, and this difference in prevalence rate is responsible for the improvement. To investigate this issue, we ran a fourth experiment in which we varied whether the probe trials included feedback or not.

### Methods

The methods were identical to the condition of Experiment 2 in which one target appeared in 80% of the probe trials, with the other target appearing in the remaining 20% of probe trials. The only difference was that we manipulated whether the probe trials provided feedback or were indistinguishable from the “real” trials. Eighty-four new subjects were randomly assigned to receive feedback or no feedback during their probe block. Of these, data from one participant were eliminated due to false alarms > 45%, and data from two were eliminated for having hit rates > 3 standard deviations from the mean hit rate, leaving a final sample size of 81 (38 in the no feedback condition).

### Results

#### Accuracy

False alarms were again exceedingly rare, < 1.8% across all conditions. A 2 (Block: Control/Experimental) × 2 (Feedback: Yes/No) mixed-model ANOVA on false alarm rates found no main effects or interactions, all *F*(1, 79) < 1, all *p* >.85, all *η*_*p*_^2^ <.001.

**Fig. 12:**
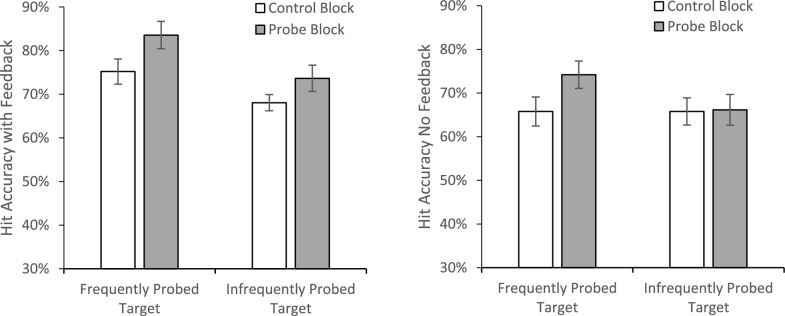
Accuracy for the feedback (left panel) and no feedback (right panel) groups as a function of target probe frequency and experimental block. Error bars depict the standard error of the mean

A 2 × 2 × 2 mixed-model ANOVA with Block (Control/Experimental) and Probe Prevalence (80%/20%) as within-subjects factors and Feedback (Yes/No) as a between-subjects factor on hit accuracy (Fig. 12) revealed a main effect of Block, *F*(1, 79) = 15.28, *p* <.001, *η*_*p*_^2^ =.16, with higher accuracy during the probe block (*M* = 74.4%, *SE* = 1.7%) than the control block (*M* = 68.7%, *SE* = 1.9%). There was also a main effect of probe prevalence, *F*(1, 79) = 10.10, *p* =.002, *η*_*p*_^2^ =.11, with higher hit rates for the frequently probed target (*M* = 74.7%, *SE* = 1.8%) than the infrequently probed target (*M* = 68.4%, *SE* = 2.0%). These two factors also interacted, *F*(1, 79) = 4.27, *p* =.042, *η*_*p*_^2^ =.05. The source of this interaction was that there were significantly more hits, *F*(1, 79) = 23.87, *p* <.001, *η*_*p*_^2^ =.23, for the frequently probed target in the probe block (*M* = 78.9%, *SE* = 1.8%) than in the control block (*M* = 70.5%, *SE* = 2.2%). However, for the infrequently probed target, hit rates in the probe (*M* = 69.9%, *SE* = 2.3%) and control (*M* = 66.9%, *SE* = 2.2%) blocks did not significantly differ, *F*(1, 79) = 1.86, *p* =.18, *η*^2^ =.02. There was also a main effect of Feedback, *F*(1, 79) = 4.95, *p* =.03, *η*_*p*_^2^ =.06, with better accuracy for subjects who received feedback in the probe trials (*M* = 75.1%, *SE* = 2.2%) than those who received no feedback (*M* = 68.0%, *SE* = 2.3%). Feedback did not interact with Block, *F*(1,79) =.79, *p* =.38, *η*_*p*_^2^ =.01, or Probe Frequency,* F*(1,79) = 1.29, *p* =.26, *η*_*p*_^2^ =.016, nor was the three-way interaction significant, *F*(1, 79) = 1.01, *p* =.32, *η*_*p*_^2^ =.013.

#### Reaction time

**Fig. 13:**
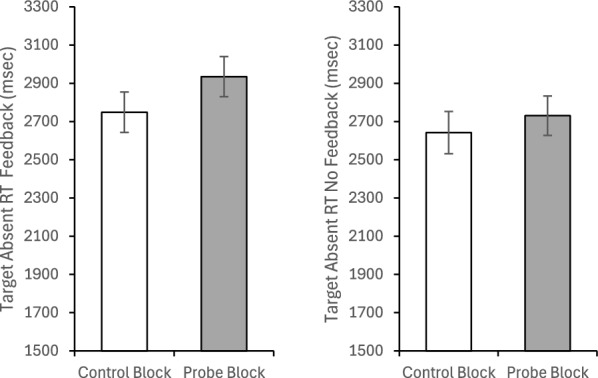
Reaction times for correct rejections for the group with feedback (left panel) and without feedback (right panel) as a function of experimental block. Error bars depict standard error of the mean

We again investigated target absent RTs as an index of quitting thresholds (Fig. 13). A 2 (Block: Control/Probe) × 2 (Feedback: Yes/No) mixed-model ANOVA on target absent RTs revealed a main effect of Block, *F*(1, 79) = 6.17, *p* =.02, *η*_*p*_^2^ =.07, with longer target absent RTs during the probe block (*M* = 2836.20, *SE* = 72.29) than the control block (*M* = 2708.96, *SE* = 75.39). There was no main effect of Feedback, *F*(1, 79) = 1.03, *p* =.31, *η*_*p*_^2^ =.01, nor did it interact with Block, *F*(1, 79) = 1.03, *p* =.31, *η*_*p*_^2^ =.01.

**Fig. 14:**
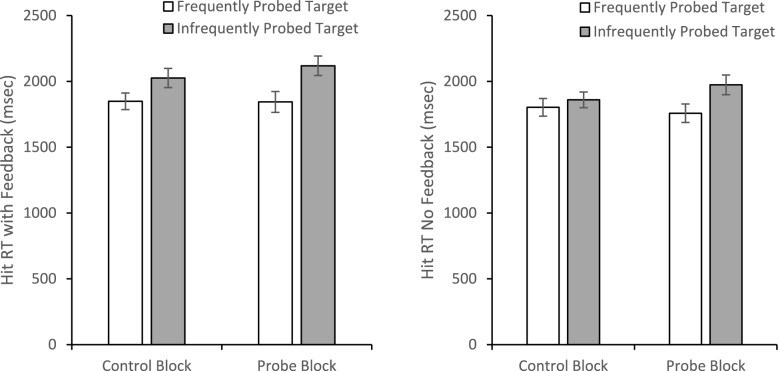
Reaction times for hits for the group with feedback (left panel) and without feedback (right panel) as a function of block and probe target frequency. Error bars depict the standard error of the mean

The same 2 × 2 × 2 mixed-model ANOVA used to evaluate accuracy was used to evaluate target present RTs (Fig. 14). There was a main effect of probe frequency, *F*(1, 79) = 19.49, *p* <.001, *η*_*p*_^2^ =.20, with faster RTs to find the frequently probed target (*M* = 1810.43, *SE* = 42.22) than the infrequently probed target (*M* = 1993.86, *SE* = 41.67). The main effect of Block was not significant, *F*(1, 79) =.94, *p* =.34, *η*_*p*_^2^ =.01; however, this was qualified by a marginally significant Block × Probe Frequency interaction, *F*(1, 79) = 3.89, *p* =.052, *η*_*p*_^2^ =.047. The source of this marginal interaction appears to be that the magnitude of the RT difference between frequently and infrequently probed targets was greater in the probe block (mean difference = 244.86 ms, *SE* = 56.1) than in the control block (*M* = 122.02 ms, *SE* = 47.4). Importantly, there was no main effect of Feedback, *F*(1, 79) = 2.18, *p* =.14, *η*_*p*_^2^ =.03, and none of the two- or three-way interactions with Feedback approached significance, all *F*(1, 79) < 1.28, all *p* >.26, all *η*_*p*_^2^ <.017.

### Discussion

The results from this experiment generally confirm earlier findings that including probe trials increases rare-target detections, especially for frequently probed targets. Accuracy was higher in the probe block than the control block, driven by higher accuracy rates for the frequently probed target. Additionally, target absent RTs were slower in the probe block than the control block, consistent with prior behavioral experiments but inconsistent with the eye tracking experiment. This increase in target absent RTs is generally interpreted as an increase in quitting thresholds. Furthermore, target present RTs were faster for frequently probed than infrequently probed targets, suggesting that attention may be guided toward the features of frequently probed targets, consistent with guidance conclusion from Experiment 3.

However, the main goal of this experiment was to determine whether feedback during probe trials is essential to the probe benefit, or whether the benefit is primarily driven by changes in perceived prevalence rates. Overall, the results suggest that the primary driver of the probe benefit is a shift in perceived prevalence. If feedback were essential, we would expect it to interact with other factors in our analyses—but it did not. Even so, providing feedback produced a significant main effect of increased accuracy, indicating that feedback is beneficial to performance.

A more nuanced inspection of the accuracy data (see Fig. 12) suggests that feedback may be particularly important for improving detection of targets that are infrequently probed. Planned paired t tests comparing accuracy in the probe block to the control block reveal that without feedback, the probe benefit occurs only for the frequently probed item, *t*(37) = 3.32, *p* =.001, *d* =.54. The infrequently probed item does not show a probe benefit, *t*(37) = 0.12, *p* =.45, *d* =.02. However, when feedback was provided, a probe benefit emerged for both the frequently probed item, *t*(41) = 3.60, *p* <.001, *d* =.55, and the infrequently probed item, *t*(42) = 1.77, *p* =.042, *d* =.27. Thus, feedback appears to be particularly beneficial for improving detection of targets that are infrequently probed.

This could be due to feedback enhancing overall motivation, which may be especially helpful for detecting low-prevalence targets. Alternatively, feedback may ensure that participants see the probed targets, allowing all probe trials to influence perceived prevalence rates. Without feedback, targets are missed in approximately 30% of probe trials, meaning those trials should not affect perceived prevalence. We cannot distinguish between these two possibilities, and both may be true.

Regardless, while probes without feedback can improve target detection (likely by altering perceived prevalence), feedback increases their effectiveness, likely by both enhancing motivation and maximizing the influence of probe trials on perceived prevalence rates.

Thus while feedback may not be required to achieve a probe benefit, from a practical perspective, if one were attempting to improve rare-target detections by including a set of probe trials with known targets, it may be beneficial to include feedback on those trials, as it ensures that the target is seen and can influence the perceived prevalence of targets.

## General discussion

Across four experiments we investigated whether interleaving a set of probe trials, ones that included a target and presented post-response feedback, would increase the detection rate for rare targets, ameliorating the low-prevalence effect. All four experiments found that the use of probes increased rare-target detection. While this overall finding suggests that probes might be used to increase rare-target detection in important real-world searches where targets are rare, the evidence from Experiments 2–4 provides some important caveats.

Specifically, in Experiment 2 when people search for either of two distinct targets, the magnitude of the benefit was directly proportional to the percentage of probe targets that matched each. This finding suggests that the probe benefit depends critically on the features of the probed targets matching those of the actual targets. Further, Experiment 3 suggests that the reason for this dependency is that the probes exert their benefit by engaging top-down guidance toward the features of the frequently probed targets. Three pieces of evidence support the involvement of this top-down mechanism. The first is that target present RTs are faster for targets that matched the frequently probed targets, and eye movement analyses find that fewer unique items need to be fixated to find the frequently probed target. The second, perhaps more direct evidence, comes from the finding in Experiment 3 that fixations during target absent trials are biased toward distractors that share features with the frequently probed target, rather than distractors that share features with the less frequently probed target. The third evidence is that there were fewer search errors, errors where the target was never fixated, for probes that matched the frequently probed targets than targets that matched the infrequently probed targets in Experiment 3. All of these findings provide compelling evidence that the probes increase top-down guidance toward objects that share features with the frequently probed items, a mechanism that would make the probe benefit selective to targets that are similar to the frequently probed targets.

Probes may also increase the trial-wide quitting thresholds, leading to more complete searches before responding target absent. Consistent with this view, in Experiments 1, 2, and 4, probes produce longer target absent reaction times, relative to control conditions that had no probes. While we do not rule out that probes may increase trial wide quitting thresholds, we do not believe that such a shift is primarily responsible for the probe benefit. Our rationale is twofold. First, in Experiment 3 we failed to find evidence for probes increasing quitting thresholds—measured in terms of either the target absent RTs or the number of unique items fixated prior to executing a target absent response. Despite no evidence for a shift in quitting thresholds in that experiment, we still find evidence for a probe benefit, suggesting that the probe benefit does not depend critically on a shift in quitting thresholds. Second, if the sole impact of probes was to increase quitting threshold, thereby resulting in more complete searches prior to a target absent response, one might expect that the probe benefit would extend to targets that did not match the probed targets—a more complete search might produce better detection of all targets. However, we find little evidence for a probe benefit for targets that never matched the probed target (Experiment 2).

In short, while probes might tend to increase quitting thresholds, our data suggest that the primary driver of the probe benefit is their engagement of top-down guidance toward features associated with frequently probed targets. Given this top-down guidance, even if probes produce an increase in trial-wide quitting thresholds, the resulting more complete searches might not benefit targets that do not share features with the probed targets. This is because the additional items scrutinized prior to executing a target absent response would likely be additional distractors that share features with the probed targets.

Experiment 4 further suggests that the benefit of probe trials arises because their inclusion alters the perceived prevalence rate of specific targets, rather than the feedback associated with probe trials directly modifying search mechanisms. While feedback during probe trials is not essential to achieve a probe benefit, we still recommend including immediate feedback, particularly for missed probes, for two reasons. First, it ensures that the target during probe trials is seen, allowing its presence to influence perceived prevalence rates (Wolfe et al., 2007). Second, feedback produced a main effect of improvement, likely due to increased motivation or vigilance. Consistent with this, research on the implementation of the TIP system found that threat detection rates increased in airports that had adopted the system compared to those that had not. Additionally, baggage screeners reported that the system made the task more engaging, leading to speculation that it enhanced vigilance (Catchpole et al., 2023). The benefit of probes being driven by top-down guidance and changes in perceived prevalence rates for specific targets is consistent with other research on the low-prevalence effect (LPE) that did not implement probes. For instance, studies that involved searching for two types of targets, one presented at a moderate prevalence rate and the other at a low-prevalence rate, have found that performance scales with the prevalence rate of each specific target (Hout et al., 2015; Wolfe et al., 2005). These findings suggest that it is not the overall prevalence rate that matters, but rather the prevalence rate of individual targets. If probes alter the perceived prevalence of specific targets, our findings align well with those prior experiments. Furthermore, it is worth noting that one of those studies used eye tracking and concluded that increasing the prevalence rate of a given target led to enhanced guidance toward that specific target (Hout et al., 2015). Thus, if our probe manipulation increases the perceived prevalence of specific targets, our finding that probes enhance search guidance fits well with previous research.

Moreover, our findings that the probe benefit results from increased guidance driven by changes in perceived prevalence rates for specific targets may have practical implications for the use of probes in real-world scenarios. Our results suggest that the benefit depends on probe trial targets sharing features with actual targets and appearing with some frequency. These requirements may limit the effectiveness of probe techniques in real-world settings. For example, in baggage screening, where threats may have highly heterogeneous features, it could be impractical to conduct frequent probe trials that encompass the full range of threat-relevant features.

Nonetheless, we believe it remains an open question to what extent the probe benefits might generalize to more complex, real-world tasks. To investigate the role of guidance, we used a highly controlled scenario with very simple stimuli. Our two targets (and their matching distractors) were designed to have no overlapping features. For instance, the T targets and their corresponding L distractors consisted of vertical and horizontal straight lines intersecting at 90 degrees, whereas the O targets and corresponding Q distractors were largely curved with no 90-degree intersections. While these maximally distinct stimuli were beneficial for evaluating the extent to which probes engaged top-down guidance, real-world search targets are likely to span a much more heterogeneous feature space, potentially increasing the number of items to which attention is biased.

In addition, while search guidance tends to improve as the search template becomes more precise (Malcolm & Henderson, 2009; Maxfield & Zelinsky, 2012; Vickery et al., 2005), there is evidence that search can be guided by semantic and categorical information (Hwang et al., 2011; Wilschut et al., 2014; Yang & Zelinsky, 2009). Based on that work, it is possible that heterogeneous probes from within a category (e.g., guns, knives, bombs) that activated a common superordinate category (e.g., weapons) might increase detection of all members of the superordinate category. However, additional research investigating the effect of probes with more complex and categorical stimuli would be needed before such claims can be substantiated.

Such work might also result in searches that are more challenging and lead to more possibilities for false alarms. In our experiments, false alarms were exceedingly rare, and thus we were unable to calculate traditional measures of sensitivity (e.g., d’) or criterion. Our observed changes in miss rates without a change in false alarms, and our finding that probes increased guidance suggests that these interventions may increase sensitivity. While there are reports of prevalence effects that are associated with changes in sensitivity (Kunar et al., 2021; Wolfe et al., 2013), the “classic” view suggests that prevalence rate primarily impacts criterion (Wolfe et al., 2005, 2007). Research with more difficult search discriminations that allowed for direct evaluation of sensitivity and criterion would help resolve how probes may impact both factors.

It is worth noting that some researchers investigating the TIP system have suggested that the optimal parameters settings of the TIP system may impact its effectiveness in improving threat detections. For instance, the composition of the image library, the timing and frequency of TIP images, and feedback about specific types of missed targets may all be critical for enhancing detection (Catchpole et al., 2023; Cutler & Paddock, 2009; Riz à Porta et al., 2022). Given that performance data on the TIP system are classified (Sensitive security information, 2025), it is difficult to ascertain the extent to which these parameters are being manipulated and studied to optimize the performance of the system. However, our data suggest that these parameters are important and optimizing them may allow the TIPs system to evolve from a system to train and evaluate operators to one that directly improves performance. While the relationship of research on the use of probes to baggage screening is clear given the existence of the TIPs system, similar methods may also improve rare-target detection in other real-world domains where missing rare targets has serious consequences, such as radiology.

## Conclusions

Here we show that one can increase the detection rate of rare targets in visual search via a method of inserting probe trials—trials that consist of a known target and provide post-response feedback. In addition, our results suggest that the probe benefit is primarily driven by increasing the perceived prevalence rate of specific targets, thereby increasing top-down guidance toward items in the display which share features with probed targets. As a result, with the simplified stimuli we used here, the magnitude of the probe benefit scales with the frequency with which the targets during the probe trials match the features of the targets during actual trials. From a practical standpoint, the fact that probes can increase rare-target detection suggests that it might be implemented to increase rare-target detection in important real-world scenarios. However, the finding that the benefit is driven by changes in top-down guidance provides important constraints on the method’s practical applicability. It will be important to establish that the probes within a real-world context are similar enough to the real targets to provide a real-world benefit.

## Data Availability

SPSS data files of the processed subject-level data for all experiments are provided on OSF at https://osf.io/uwbqx/

## Footnotes

1 We choose to separately calculate Hits and False Alarms rather than calculate d’ because false alarms were so rare, that d’ analyses would be extremely influenced by the convention we used to calculate it when there were zero false alarms.

## Code availability

SPSS syntax for all the comparisons in the manuscript is available at https://osf.io/uwbqx/. Open Practices Statement: The experiments were not preregistered, but the data and analysis syntax are publicly available at https://osf.io/uwbqx/.
